# (4-Nitro­phen­yl)methyl 2,3-di­hydro-1*H*-pyrrole-1-carboxyl­ate: crystal structure and Hirshfeld analysis

**DOI:** 10.1107/S2056989018002451

**Published:** 2018-02-23

**Authors:** Julio Zukerman-Schpector, Monica Soto-Monsalve, Regina H. De Almeida Santos, Ariel L. L. Garcia, Carlos Roque D. Correia, Mukesh M. Jotani, Edward R. T. Tiekink

**Affiliations:** a Laboratório de Cristalografia, Esterodinâmica e Modelagem Molecular, Departamento de Química, Universidade Federal de São Carlos, 13565-905 São Carlos, SP, Brazil; b Instituto de Química de São Carlos, Universidade de São Paulo, São Carlos, SP, Brazil; c Instituto de Química, Universidade Estadual de Campinas, UNICAMP, CP 6154, CEP. 13084-971, Campinas, São Paulo, Brazil; d Department of Physics, Bhavan’s Sheth R. A. College of Science, Ahmedabad, Gujarat 380 001, India; e Centre for Crystalline Materials, School of Science and Technology, Sunway University, 47500 Bandar Sunway, Selangor Darul Ehsan, Malaysia

**Keywords:** crystal structure, di­hydro­pyrrole, ester, nitro-O⋯π inter­actions, Hirshfeld surface analysis

## Abstract

In the title compound, C_12_H_12_N_2_O_4_, the di­hydro­pyrrole ring is almost planar (r.m.s. deviation = 0.0049 Å) and is nearly coplanar with the adjacent C_2_O_2_ residue [dihedral angle = 4.56 (9)°], which links to the 4-nitro­benzene substituent [dihedral angle = 4.58 (8)°]. The mol­ecule is concave, with the outer rings lying to the same side of the central C_2_O_2_ residue and being inclined to each other [dihedral angle = 8.30 (7)°]. In the crystal, supra­molecular layers parallel to (10-5) are sustained by nitro­benzene-C—H⋯O(carbon­yl) and pyrrole-C—H⋯O(nitro) inter­actions. The layers are connected into a three-dimensional architecture by π(pyrrole)–π(nitro­benzene) stacking [inter-centroid separation = 3.7414 (10) Å] and nitro-O⋯π(pyrrole) inter­actions.

## Abstract 2

The title compound has a ‘banana’ shape, with the dihedral angle formed by the outer rings being 8.30 (7)°. In the crystal, the three-dimensional architecture features nitro­benzene-C—H⋯O(carbon­yl), pyrrole-C—H⋯O(nitro), π(pyrrole)—π(nitro­benzene) and nitro-O⋯π(pyrrole) inter­actions.

## Chemical context   

Many hy­droxy­lated prolines and homoprolines have the ability to inhibit glycosides and glycosyl­transferases, key enzymes in biosynthesis and the processing of glycoproteins and glycolipids (Rule *et al.*, 1985 ▸; Fleet & Son, 1988 ▸; Wong, 1997 ▸). Glycoproteins are macromolecules involved in the recognition (cell–cell inter­actions and host–pathogen) and control of mechanisms associated with biological structures. Thus, compounds that are capable of inhibiting the biosynthetic pathway of glycoproteins have broad chemotherapeutic potential in the treatment of metabolic diseases such as diabetes, obesity, cancer, tuberculosis and viral infections among others (Kordik & Reitz, 1999 ▸; Nishimura, 2003 ▸; Cheng & Josse, 2004 ▸). Some hy­droxy­lated prolines are of inter­est in this context owing to their ability to inhibit glycosidases and because they are found as substructures of natural bioactive compounds. For example, (2*S*,3*R*,4*S*)-3,4-di­hydroxy­proline (II), see scheme[Chem scheme1], is found as a component of the repeating deca­peptide sequence of the Mefp1 adhesive protein (Mytilus edulis foot protein 1), produced by the marine mussel, Mytilus edulis (Taylor *et al.*, 1994 ▸; Taylor & Weir, 2000 ▸). This protein is responsible for the fixation of mussels to rocks. As a part of a study into the development of new and flexible methodologies for the efficient synthesis of several natural and synthetic products with important pharmacological properties, using the Heck–Matsuda aryl­ation reaction as a crucial step, (II) was prepared from the title compound, (I), for the purpose of evaluating the best protecting group for use in future syntheses of greater complexity (Garcia, 2008 ▸). During the Heck–Matsuda reaction, it was found that the protective group of the nitro­gen atom in (I) exerted some influence on the reaction time, but did not influence the yield of the expected inter­mediate when compared to the Heck–Matsuda reaction applied to the enecarbamate, ethyl 2,3-di­hydro-1*H*-pyrrole-1-carboxyl­ate (Garcia, 2008 ▸). It is noted that the first synthesis of (I) was actually reported nearly 50 years ago (Heine & Mente, 1971 ▸). Herein, the crystal and mol­ecular structures of (I) are described along with an analysis of the calculated Hirshfeld surfaces.
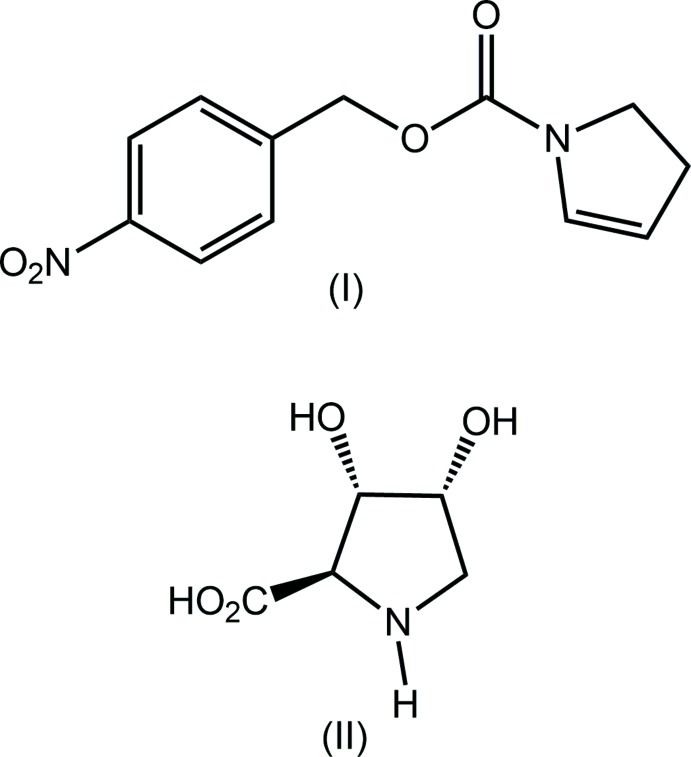


## Structural commentary   

**Figure 1:**
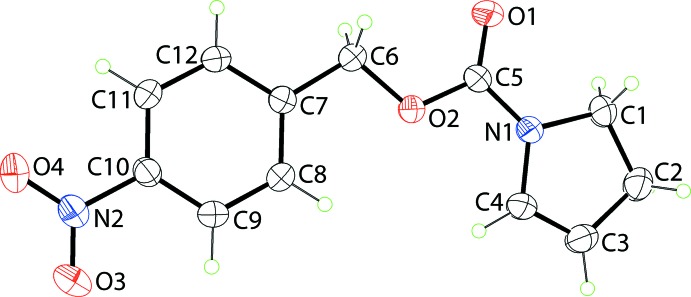
The mol­ecular structure of (I), showing the atom-labelling scheme and displacement ellipsoids at the 35% probability level.

The mol­ecular structure of (I), Fig. 1 ▸, is a 1-methyl­ene-4-nitro­benzene ester derived from di­hydro­pyrrole-1-carb­oxy­lic acid. In (I), the di­hydro­pyrrole ring is almost planar with the r.m.s. deviation of the five fitted atoms being 0.0049 Å, and the maximum deviation of any of the constituent atoms being 0.0065 (11) Å for atom C2. The adjacent C_2_O_2_ residue (O1,O2,C5,C6) is essentially co-planar, with the dihedral angle between the two planes being 4.56 (9)°. The planarity extends to the 4-nitro­benzene ring, with the dihedral angle between the C_2_O_2_ and C_6_ planes being 4.58 (8)°. However, the mol­ecule is not planar but rather is curved as the outer rings lie to the same side of the central C_2_O_2_ residue; the dihedral angle = 8.30 (7)°. To a first approximation, the nitro group is co-planar with the benzene ring to which is connected, as seen in the value of the O4—N2—C10—C9 torsion angle of 173.50 (15)°.

## Supra­molecular features   

**Figure 2:**
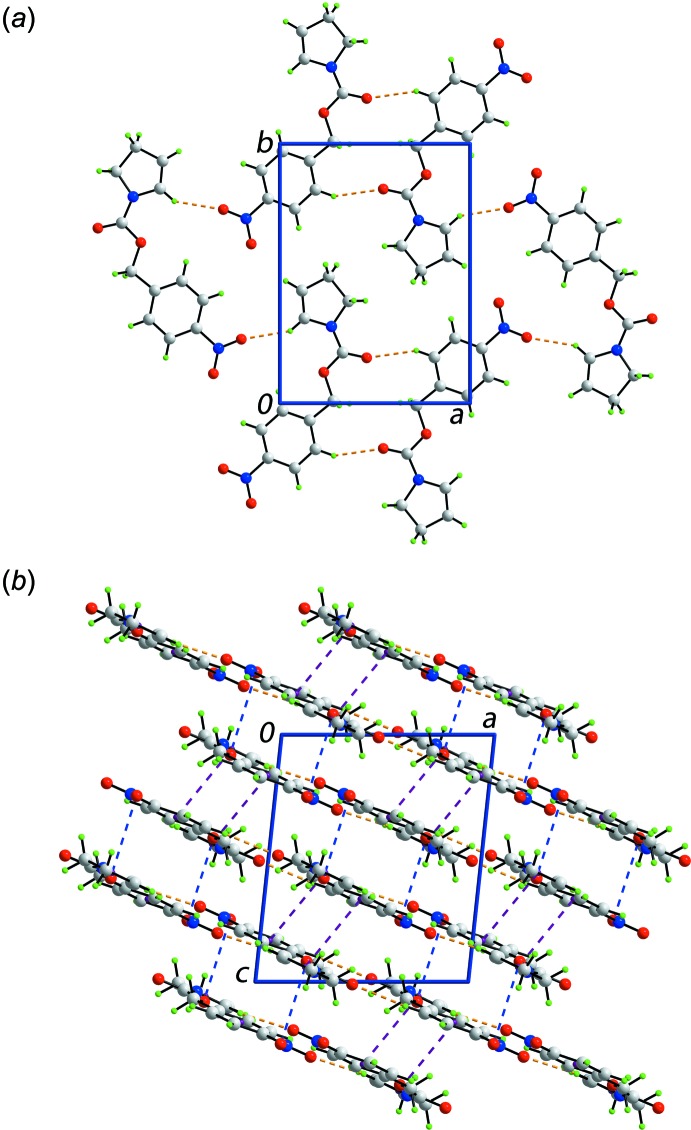
Mol­ecular packing in (I): (*a*) view of the supra­molecular layer parallel to (10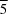) plane and (*b*) view of the unit-cell contents shown in projection down the *b* axis. The C—H⋯O, N—O⋯π and π–π contacts are shown as orange, blue and purple dashed lines, respectively.

The mol­ecular packing of (I) features a variety of directional inter­actions, Table 1 ▸. Thus, nitro­benzene-C12—H⋯O1(carbon­yl) inter­actions occur over a centre of inversion and lead to 14-membered {⋯HC_3_OCO}_2_ synthons. The dimeric aggregates are connected into a supra­molecular layer *via* pyrrole-C4—H⋯O3(nitro) inter­actions. The layers lie parallel to (10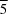), Fig. 2 ▸ *a*. Two types of inter­actions connect layers into a three-dimensional architecture.Thus, π(N1,C1–C4)–π(C7–C12)^i^ stacking inter­actions occur between pyrrole and nitro­benzene rings: inter-centroid separation = 3.7414 (10) Å and angle of inclination = 7.99 (9)° for symmetry code: (i): 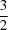 − *x*, −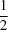 + *y*, 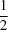 − *z*. The other inter­actions between layers are of the type nitro-O4⋯π(N1,C1–C4), Table 1 ▸. These inter­actions are well known in consolidating the packing of nitro-containing compounds (Huang *et al.*, 2008 ▸). A view of the unit-cell contents is shown in Fig. 2 ▸ *b*.

## Hirshfeld surface analysis   

The Hirshfeld surface calculations for (I) were performed as per a recent study (Zukerman-Schpector *et al.*, 2017 ▸) and serve to provide additional information on the mol­ecular packing.

**Figure 3:**
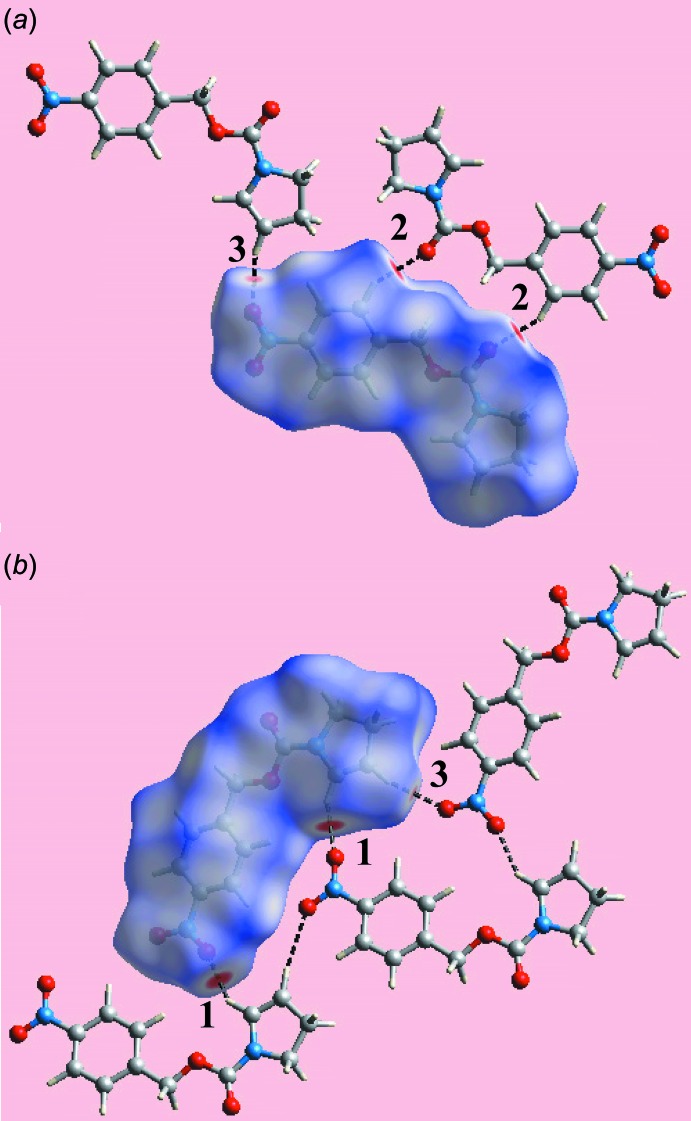
Two views of the Hirshfeld surface for (I) mapped over *d*_norm_ in the range −0.225 to +1.393 au, showing inter­molecular C—H⋯O contacts as black dashed lines.

**Figure 4:**
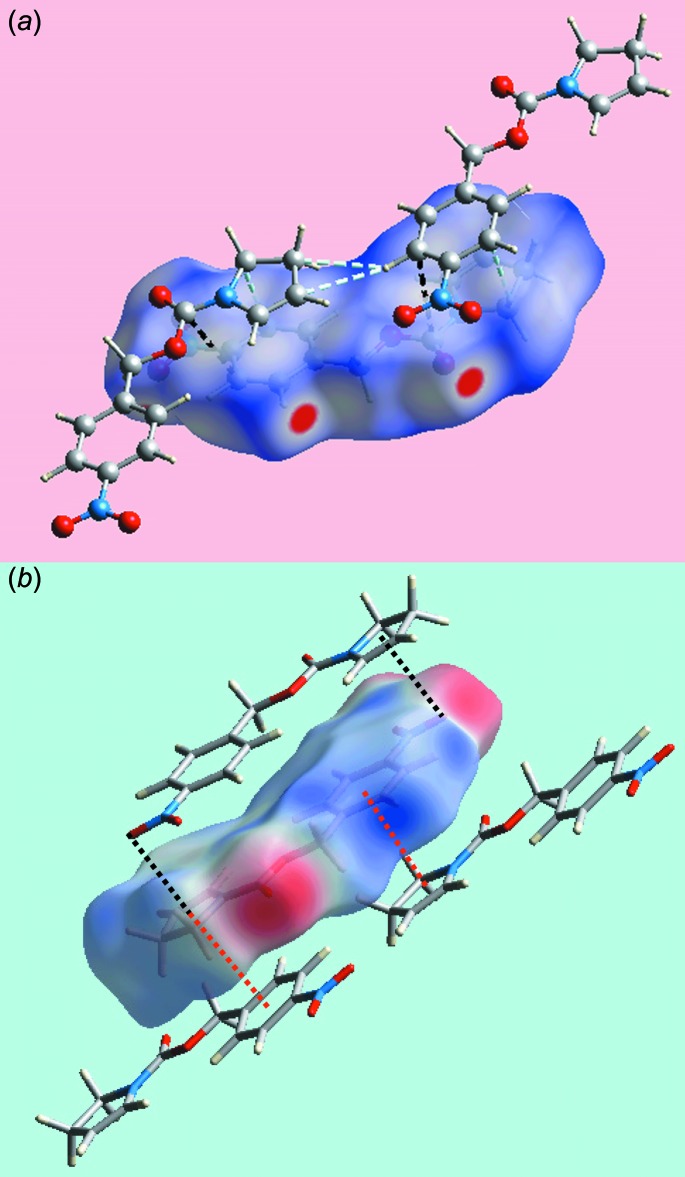
Views of Hirshfeld surfaces for (I) mapped: (*a*) over *d*_norm_ in the range −0.225 to + 1.393 au, highlighting inter- and intra-layer C⋯C and C⋯H/H⋯C contacts as black and sky-blue dashed lines, respectively, and (*b*) over the electrostatic potential in the range ±0.077 au (the red and blue regions represent negative and positive electrostatic potentials, respectively), showing inter­molecular N—O⋯π and π–π contacts as black dotted lines.

In addition to the bright-red spots on the Hirshfeld surface mapped over *d*_norm_ in Fig. 3 ▸ near the pyrrole-H4, nitro­benzene-H12, and the nitro-O3 and carbonyl-O1 atoms, representing the respective donors and acceptors of inter­molecular C—H⋯O inter­actions (labelled ‘1’ and ‘2’), the diminutive red spots appearing near the pyrrole-H3 and nitro-O4 atoms in Fig. 3 ▸ (labelled ‘3’) also indicate the influence of comparatively weak C—H⋯O contacts in the crystal (Table 2 ▸). The nitro­benzene-C9 and C11 atoms form inter-layer short C⋯H/H⋯C and C⋯C contacts (Table 2 ▸) with the pyrrole-H1*B* and ester-C5 atoms, respectively, Fig. 4 ▸ *a*. The other short inter­atomic C⋯H/H⋯C contacts between the nitro­benzene-H11 and pyrrole-C2 and C3 atoms (Table 2 ▸) are intra-layer, Fig. 4 ▸ *a*. The building up of the three-dimensional architecture through π–π-stacking inter­actions and nitro-N—O⋯π(pyrrole) contacts is highlighted in Fig. 4 ▸ *b*, showing the Hirshfeld surface mapped over the electrostatic potential.

**Figure 5:**
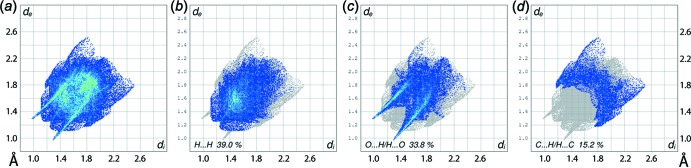
(*a*) The full two-dimensional fingerprint plot for (I) and those delineated into (*b*) H⋯H, (*c*) O⋯H/H⋯O and (*d*) C⋯H/H⋯C contacts.

The overall two-dimensional fingerprint plot and those delineated into H⋯H, O⋯H/H⋯O and C⋯H/H⋯C contacts (McKinnon *et al.*, 2007 ▸) are illustrated in Fig. 5 ▸ *a*–*d*, respectively, and the percentage contribution from the identified inter­atomic contacts to the Hirshfeld surface are summarized in Table 3 ▸. The comparatively low, *i.e*. 39.0%, contribution from H⋯H contacts to the overall surface is due to the involvement of many hydrogen atoms in directional inter­molecular inter­actions, *e.g*. C—H⋯O, π (Tables 1 ▸ and 2 ▸). Hence, the inter­atomic H⋯H contacts have a reduced influence in the crystal as their inter­atomic separations are equal to or greater than sum of their van der Waals radii (Fig. 5 ▸ *b*). Conversely, the relatively significant contribution of 33.8% from O⋯H/H⋯O contacts to the Hirshfeld surface is consistent with this observation. The fingerprint plot delineated into O⋯H/H⋯O contacts (Fig. 5 ▸ *c*) features a pair of green aligned points within the pair of spikes with their tips at *d*_e_ + *d*_i_ ∼2.3 Å superimposed upon a distribution blue points characterizing inter­molecular C—H⋯O inter­actions. The short inter­atomic C⋯H/H⋯C contacts in the inter- and intra-layer regions are represented by the two pairs of short forceps-like spikes at *d*_e_ + *d*_i_ ∼2.8 and 2.9 Å, respectively, in Fig. 5 ▸ *d*. The small but discernible contributions from inter­atomic C⋯C and C⋯N/N⋯C contacts (Table 3 ▸) result from short inter-layer contacts and π–π stacking inter­actions. The presence of the N—O⋯π contact in the structure is also evident from the contribution of C⋯O/O⋯C and N⋯O/O⋯N contacts to the Hirshfeld surface as summarized in Table 3 ▸. The small contributions from the other remaining inter­atomic contacts (Table 3 ▸) have a negligible influence on the packing.

## Database survey   

Di­hydro­pyrrole rings as found in (I) have rarely been characterized crystallographically and only one structure is deposited in the Cambridge Structural Database (Groom *et al.*, 2016 ▸), namely the adduct, ZnI_2_(4,5-di­hydro-3*H*-pyrrole)_2_ (refcode WAZXAW; Freer *et al.*, 1993 ▸). Here, despite having *sp* ^2^-carbon centres as in (I), the rings are planar with one lying on a crystallographic mirror plane and the other disposed across a mirror plane (r.m.s. deviation = 0.007 Å), implying disorder in the latter.

## Synthesis and crystallization   

A solution of (4-nitro­phen­yl)methyl 2-hy­droxy­pyrrolidine-1-carboxyl­ate (2.85 g, 10.704 mmol) in toluene (100 ml) was cooled to 273 K in an ice/water bath. Under an atmosphere of nitro­gen, 2,4-lutidine (6.2 ml, 53.634 mmol) was added to this solution. The solution was stirred for 15 min at 273 K. A tri­fluoro­acetic anhydride (TFAA) solution (13.2 ml of a 0.8 *M* solution, 10.56 mmol) in dry toluene was then added. The bath was removed and the solution stirred for 2 h at room temperature. Subsequently, the flask was immersed for 20 min in an oil bath preheated to 393–403 K with a reflux condenser. The solution was concentrated in a rotary evaporator and the residue was purified by flash column chromatography on silica gel, using a mixture of EtOAc/*n*-hexane (1:4) as the eluent. The yield of (I) was 2.103 g (80% based on TFAA). Irregular yellow crystals of (I) were obtained from the slow evaporation of its CH_2_Cl_2_ solution.

Spectroscopic characterization. ^1^H NMR (300 MHz, Py-*d*_5_, solution comprises rotamers): δ 8.21 (apparent *d*, *J* = 7.3 Hz, 2H, H3′ and H5′), 7.54 (*d*, *J* = 8.1 Hz, 2H, H2′ and H6′), 6.80 and 6.68 (2 × *m*, 1H, H2), 5.35 (*s*, 2H, CH_2_), 5.03 (*m*, 1H, H3), 3.71 (apparent *t*, *J* = 9.5 Hz, 2H, H5*a*,5*b*), 2.46 (apparent quint., *J* = 9.5 Hz, 2H, H4*a*,4*b*). ^13^C NMR (75 MHz, Py-*d*_5_, solution comprises rotamers): δ = 152.3 (CO_2_ *R*), 151.5 (CO_2_ *R*), 147.8 (C4′), 144.9 (C1′), 129.8 (C2), 129.2 (C2), 128.4 (C2′ and C6′), 128.3 (C2′ and C6′), 123.9 (C3′ and C5′), 109.4 (C3), 65.8 (CH_2_), 65.6 (CH_2_), 45.8 (C5), 45.4 (C5), 30.1 (C4), 29.0 (C4). ESI–MS (*m*/*z*) calculated for C_12_H_12_N_2_O_4_ 248.07971, found 248.07876.

## Refinement details   

Crystal data, data collection and structure refinement details are summarized in Table 4 ▸. The carbon-bound H atoms were placed in calculated positions (C—H = 0.93–0.97 Å) and were included in the refinement in the riding-model approximation, with *U*_iso_(H) set to 1.2*U*_eq_(C).

## Supplementary Material

Crystal structure: contains datablock(s) I, global. DOI: 10.1107/S2056989018002451/hb7736sup1.cif

Structure factors: contains datablock(s) I. DOI: 10.1107/S2056989018002451/hb7736Isup2.hkl

Supporting information file. DOI: 10.1107/S2056989018002451/hb7736Isup3.cml

CCDC reference: 1823263

Additional supporting information:  crystallographic information; 3D view; checkCIF report

## Figures and Tables

**Table 1:** Hydrogen-bond geometry (Å, °) *Cg*1 is the centroid of the N1/C1–C4 ring.

*D*—H⋯*A*	*D*—H	H⋯*A*	*D*⋯*A*	*D*—H⋯*A*
C4—H4⋯O3^i^	0.93	2.40	3.227 (2)	149
C12—H12⋯O1^ii^	0.93	2.47	3.318 (2)	152
N2—O4⋯*Cg*1^iii^	1.22 (1)	3.42 (1)	3.6327 (16)	90 (1)

Symmetry codes: (i) 
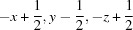
; (ii) 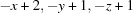; (iii) 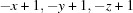.

**Table 2:** Summary of short inter­atomic contacts (Å) in (I)

Contact	Distance	Symmetry operation
O4⋯H3	2.47	*x*, −1 + *y*, *z*
C5⋯C11	3.37	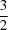 − *x*, − 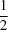 + *y*, 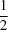 − *z*
C2⋯H11	2.81	*x*, −1 + *y*, *z*
C3⋯H11	2.91	*x*, − 1 + *y*, *z*
C9⋯H1*B*	2.92	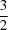 − *x*, 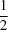 + *y*, 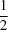 − *z*

**Table 3:** Percentage contributions of inter­atomic contacts to the Hirshfeld surface for (I)

Contact	Percentage contribution
H⋯H	39.0
O⋯H/H⋯O	33.8
C⋯H/H⋯C	15.2
C⋯O/O⋯C	3.7
C⋯C	2.4
C⋯N/N⋯C	1.7
O⋯O	1.4
N⋯H/H⋯N	1.0
N⋯O/O⋯N	0.9
N⋯N	0.9

**Table 4:** Experimental details

Crystal data	
Chemical formula	C_12_H_12_N_2_O_4_
*M*_r_	248.24
Crystal system, space group	Monoclinic, *P*2_1_/*n*
Temperature (K)	290
*a*, *b*, *c* (Å)	9.0385 (3), 12.2518 (4), 10.5452 (3)
β (°)	96.102 (1)
*V* (Å^3^)	1161.14 (6)
*Z*	4
Radiation type	Mo *K*α
μ (mm^−1^)	0.11
Crystal size (mm)	0.52 × 0.22 × 0.14

Data collection	
Diffractometer	Bruker APEXII CCD
Absorption correction	Multi-scan (*SADABS*; Sheldrick, 1995 ▸)
*T*_min_, *T*_max_	0.724, 0.745
No. of measured, independent and observed [*I* > 2σ(*I*)] reflections	23727, 2394, 2013
*R*_int_	0.023
(sin θ/λ)_max_ (Å^−1^)	0.627

Refinement	
*R*[*F* ^2^ > 2σ(*F* ^2^)], *wR*(*F* ^2^), *S*	0.041, 0.117, 1.09
No. of reflections	2394
No. of parameters	163
H-atom treatment	H-atom parameters constrained
Δρ_max_, Δρ_min_ (e Å^−3^)	0.16, −0.18

Computer programs: *APEX2* and *SAINT* (Bruker, 2009 ▸), *SIR2014* (Burla *et al.*, 2015 ▸), *SHELXL2014* (Sheldrick, 2015 ▸), *ORTEP-3 for Windows* (Farrugia, 2012 ▸), *DIAMOND* (Brandenburg, 2006 ▸), *MarvinSketch* (ChemAxon, 2010 ▸) and *publCIF* (Westrip, 2010 ▸).

## supplementary crystallographic information

### Crystal data

**Table:**

C_12_H_12_N_2_O_4_	*F*(000) = 520
*M**_r_* = 248.24	*D*_x_ = 1.420 Mg m^−^^3^
Monoclinic, *P*2_1_/*n*	Mo *K*α radiation, λ = 0.71073 Å
*a* = 9.0385 (3) Å	Cell parameters from 9984 reflections
*b* = 12.2518 (4) Å	θ = 2.6–26.5°
*c* = 10.5452 (3) Å	µ = 0.11 mm^−^^1^
β = 96.102 (1)°	*T* = 290 K
*V* = 1161.14 (6) Å^3^	Irregular, yellow
*Z* = 4	0.52 × 0.22 × 0.14 mm

### Data collection

**Table:**

Bruker APEXII CCD diffractometer	2013 reflections with *I* > 2σ(*I*)
φ and ω scans	*R*_int_ = 0.023
Absorption correction: multi-scan (SADABS; Sheldrick, 1995)	θ_max_ = 26.5°, θ_min_ = 2.6°
*T*_min_ = 0.724, *T*_max_ = 0.745	*h* = −9→11
23727 measured reflections	*k* = −15→15
2394 independent reflections	*l* = −13→13

### Refinement

**Table:**

Refinement on *F*^2^	0 restraints
Least-squares matrix: full	Hydrogen site location: inferred from neighbouring sites
*R*[*F*^2^ > 2σ(*F*^2^)] = 0.041	H-atom parameters constrained
*wR*(*F*^2^) = 0.117	*w* = 1/[σ^2^(*F*_o_^2^) + (0.0501*P*)^2^ + 0.3399*P*] where *P* = (*F*_o_^2^ + 2*F*_c_^2^)/3
*S* = 1.09	(Δ/σ)_max_ < 0.001
2394 reflections	Δρ_max_ = 0.16 e Å^−^^3^
163 parameters	Δρ_min_ = −0.18 e Å^−^^3^

### Special details

**Table:**

Geometry. All esds (except the esd in the dihedral angle between two l.s. planes) are estimated using the full covariance matrix. The cell esds are taken into account individually in the estimation of esds in distances, angles and torsion angles; correlations between esds in cell parameters are only used when they are defined by crystal symmetry. An approximate (isotropic) treatment of cell esds is used for estimating esds involving l.s. planes.

### Fractional atomic coordinates and isotropic or equivalent isotropic displacement parameters (Å^2^)

**Table:**

	*x*	*y*	*z*	*U*_iso_*/*U*_eq_	
O1	0.95640 (13)	0.32527 (10)	0.51064 (14)	0.0665 (4)	
O2	0.73098 (11)	0.38092 (8)	0.41873 (11)	0.0489 (3)	
O3	0.20267 (15)	0.75353 (13)	0.19648 (17)	0.0896 (5)	
O4	0.34938 (16)	0.88645 (10)	0.24771 (15)	0.0720 (4)	
N1	0.77129 (13)	0.20438 (10)	0.45899 (13)	0.0468 (3)	
N2	0.32222 (15)	0.78902 (12)	0.24108 (13)	0.0532 (3)	
C1	0.86033 (17)	0.10688 (13)	0.49447 (17)	0.0514 (4)	
H1A	0.8888	0.1046	0.5858	0.062*	
H1B	0.9495	0.1052	0.4508	0.062*	
C2	0.75720 (19)	0.01184 (14)	0.45210 (19)	0.0581 (4)	
H2A	0.7987	−0.0322	0.3881	0.070*	
H2B	0.7401	−0.0342	0.5239	0.070*	
C3	0.61637 (18)	0.06612 (14)	0.39787 (18)	0.0558 (4)	
H3	0.5308	0.0292	0.3652	0.067*	
C4	0.62960 (16)	0.17304 (13)	0.40258 (16)	0.0505 (4)	
H4	0.5549	0.2216	0.3725	0.061*	
C5	0.83046 (16)	0.30513 (12)	0.46670 (15)	0.0443 (3)	
C6	0.78181 (17)	0.49186 (12)	0.42996 (17)	0.0490 (4)	
H6A	0.8665	0.5019	0.3819	0.059*	
H6B	0.8127	0.5089	0.5186	0.059*	
C7	0.65706 (15)	0.56621 (11)	0.37923 (13)	0.0400 (3)	
C8	0.51427 (16)	0.52938 (12)	0.33957 (14)	0.0442 (3)	
H8	0.4927	0.4553	0.3436	0.053*	
C9	0.40373 (17)	0.60201 (12)	0.29406 (15)	0.0445 (3)	
H9	0.3082	0.5774	0.2672	0.053*	
C10	0.43818 (16)	0.71143 (12)	0.28935 (13)	0.0414 (3)	
C11	0.57881 (17)	0.75032 (12)	0.32932 (16)	0.0481 (4)	
H11	0.5996	0.8246	0.3262	0.058*	
C12	0.68753 (17)	0.67727 (12)	0.37385 (16)	0.0478 (4)	
H12	0.7828	0.7025	0.4007	0.057*	

### Atomic displacement parameters (Å^2^)

**Table:**

	*U*^11^	*U*^22^	*U*^33^	*U*^12^	*U*^13^	*U*^23^
O1	0.0418 (6)	0.0487 (7)	0.1027 (10)	−0.0039 (5)	−0.0220 (6)	0.0065 (6)
O2	0.0409 (6)	0.0331 (5)	0.0694 (7)	0.0014 (4)	−0.0086 (5)	0.0017 (4)
O3	0.0510 (8)	0.0752 (10)	0.1334 (14)	0.0052 (7)	−0.0338 (8)	0.0061 (9)
O4	0.0699 (9)	0.0433 (7)	0.0990 (10)	0.0135 (6)	−0.0083 (7)	0.0061 (6)
N1	0.0352 (6)	0.0369 (6)	0.0660 (8)	0.0025 (5)	−0.0052 (5)	0.0040 (6)
N2	0.0467 (8)	0.0498 (8)	0.0610 (8)	0.0086 (6)	−0.0039 (6)	0.0025 (6)
C1	0.0442 (8)	0.0408 (8)	0.0681 (10)	0.0078 (6)	0.0017 (7)	0.0066 (7)
C2	0.0561 (10)	0.0417 (8)	0.0766 (11)	0.0010 (7)	0.0070 (8)	0.0059 (8)
C3	0.0440 (8)	0.0476 (9)	0.0752 (11)	−0.0077 (7)	0.0032 (8)	−0.0010 (8)
C4	0.0342 (8)	0.0459 (8)	0.0696 (10)	−0.0006 (6)	−0.0026 (7)	0.0024 (7)
C5	0.0363 (7)	0.0403 (8)	0.0545 (8)	0.0023 (6)	−0.0034 (6)	0.0027 (6)
C6	0.0432 (8)	0.0352 (7)	0.0660 (10)	−0.0034 (6)	−0.0066 (7)	0.0011 (7)
C7	0.0387 (7)	0.0369 (7)	0.0435 (7)	0.0005 (6)	−0.0006 (6)	−0.0008 (6)
C8	0.0447 (8)	0.0347 (7)	0.0519 (8)	−0.0042 (6)	−0.0009 (6)	0.0008 (6)
C9	0.0367 (7)	0.0434 (8)	0.0516 (8)	−0.0046 (6)	−0.0028 (6)	−0.0015 (6)
C10	0.0391 (7)	0.0406 (8)	0.0434 (7)	0.0050 (6)	−0.0007 (6)	−0.0006 (6)
C11	0.0461 (8)	0.0325 (7)	0.0640 (9)	−0.0023 (6)	−0.0019 (7)	−0.0013 (6)
C12	0.0370 (8)	0.0392 (8)	0.0647 (9)	−0.0040 (6)	−0.0056 (7)	−0.0029 (7)

### Geometric parameters (Å, º)

**Table:**

O1—C5	1.2080 (18)	C3—H3	0.9300
O2—C5	1.3527 (17)	C4—H4	0.9300
O2—C6	1.4356 (17)	C6—C7	1.503 (2)
O3—N2	1.2123 (18)	C6—H6A	0.9700
O4—N2	1.2193 (18)	C6—H6B	0.9700
N1—C5	1.3442 (19)	C7—C8	1.389 (2)
N1—C4	1.4070 (18)	C7—C12	1.391 (2)
N1—C1	1.4665 (18)	C8—C9	1.385 (2)
N2—C10	1.4656 (19)	C8—H8	0.9300
C1—C2	1.528 (2)	C9—C10	1.378 (2)
C1—H1A	0.9700	C9—H9	0.9300
C1—H1B	0.9700	C10—C11	1.381 (2)
C2—C3	1.495 (2)	C11—C12	1.374 (2)
C2—H2A	0.9700	C11—H11	0.9300
C2—H2B	0.9700	C12—H12	0.9300
C3—C4	1.316 (2)		
			
C5—O2—C6	115.15 (11)	O1—C5—O2	124.40 (14)
C5—N1—C4	127.92 (13)	N1—C5—O2	111.31 (12)
C5—N1—C1	121.91 (12)	O2—C6—C7	108.85 (12)
C4—N1—C1	109.61 (12)	O2—C6—H6A	109.9
O3—N2—O4	122.62 (15)	C7—C6—H6A	109.9
O3—N2—C10	118.50 (14)	O2—C6—H6B	109.9
O4—N2—C10	118.88 (14)	C7—C6—H6B	109.9
N1—C1—C2	104.18 (12)	H6A—C6—H6B	108.3
N1—C1—H1A	110.9	C8—C7—C12	119.16 (13)
C2—C1—H1A	110.9	C8—C7—C6	123.24 (13)
N1—C1—H1B	110.9	C12—C7—C6	117.59 (12)
C2—C1—H1B	110.9	C9—C8—C7	120.57 (14)
H1A—C1—H1B	108.9	C9—C8—H8	119.7
C3—C2—C1	103.94 (13)	C7—C8—H8	119.7
C3—C2—H2A	111.0	C10—C9—C8	118.68 (13)
C1—C2—H2A	111.0	C10—C9—H9	120.7
C3—C2—H2B	111.0	C8—C9—H9	120.7
C1—C2—H2B	111.0	C9—C10—C11	121.93 (14)
H2A—C2—H2B	109.0	C9—C10—N2	119.17 (13)
C4—C3—C2	110.99 (14)	C11—C10—N2	118.90 (13)
C4—C3—H3	124.5	C12—C11—C10	118.75 (14)
C2—C3—H3	124.5	C12—C11—H11	120.6
C3—C4—N1	111.26 (14)	C10—C11—H11	120.6
C3—C4—H4	124.4	C11—C12—C7	120.90 (13)
N1—C4—H4	124.4	C11—C12—H12	119.5
O1—C5—N1	124.29 (13)	C7—C12—H12	119.5
			
C5—N1—C1—C2	−171.59 (15)	O2—C6—C7—C12	−175.74 (14)
C4—N1—C1—C2	0.51 (18)	C12—C7—C8—C9	0.6 (2)
N1—C1—C2—C3	−0.98 (18)	C6—C7—C8—C9	179.78 (14)
C1—C2—C3—C4	1.2 (2)	C7—C8—C9—C10	−0.2 (2)
C2—C3—C4—N1	−0.9 (2)	C8—C9—C10—C11	−0.4 (2)
C5—N1—C4—C3	171.74 (16)	C8—C9—C10—N2	179.85 (13)
C1—N1—C4—C3	0.2 (2)	O3—N2—C10—C9	−6.2 (2)
C4—N1—C5—O1	−175.78 (17)	O4—N2—C10—C9	173.50 (15)
C1—N1—C5—O1	−5.2 (3)	O3—N2—C10—C11	174.03 (17)
C4—N1—C5—O2	4.1 (2)	O4—N2—C10—C11	−6.2 (2)
C1—N1—C5—O2	174.65 (14)	C9—C10—C11—C12	0.6 (2)
C6—O2—C5—O1	−3.7 (2)	N2—C10—C11—C12	−179.62 (14)
C6—O2—C5—N1	176.46 (13)	C10—C11—C12—C7	−0.2 (2)
C5—O2—C6—C7	−176.99 (13)	C8—C7—C12—C11	−0.4 (2)
O2—C6—C7—C8	5.1 (2)	C6—C7—C12—C11	−179.60 (15)

### Hydrogen-bond geometry (Å, º)

Cg1 is the centroid of the N1/C1–C4 ring.

**Table:**

*D*—H···*A*	*D*—H	H···*A*	*D*···*A*	*D*—H···*A*
C4—H4···O3^i^	0.93	2.40	3.227 (2)	149
C12—H12···O1^ii^	0.93	2.47	3.318 (2)	152
N2—O4···*Cg*1^iii^	1.22 (1)	3.42 (1)	3.6327 (16)	90 (1)

Symmetry codes: (i) −*x*+1/2, *y*−1/2, −*z*+1/2; (ii) −*x*+2, −*y*+1, −*z*+1; (iii) −*x*+1, −*y*+1, −*z*+1.
